# Identification and validation of a novel major QTL for harvest index in rice (*Oryza sativa* L.)

**DOI:** 10.1186/s12284-017-0183-0

**Published:** 2017-09-26

**Authors:** Shaohong Zhang, Xiuying He, Junliang Zhao, Yongsheng Cheng, Zhimei Xie, Yuehan Chen, Tifeng Yang, Jingfang Dong, Xiaofei Wang, Qing Liu, Wei Liu, Xingxue Mao, Hua Fu, Zhaoming Chen, Yaoping Liao, Bin Liu

**Affiliations:** 1 Rice Research Institute, Guangdong Academy of Agricultural Sciences, Guangzhou, 510640 China; 2 Guangdong Key Laboratory of New Technology in Rice Breeding, Guangzhou, 510640 China; 3 Rice Research Institute, Hunan Agricultural University, Changsha, 410128 China; 4 Present address: Yahua Seed Science Research Institute, Longping High-tech, Changsha, 410116 China

**Keywords:** Rice (*Oryza sativa* L.), Harvest index, Grain yield, Biomass, Quantitative trait locus (QTL), Mapping, Validation

## Abstract

**Background:**

Harvest index (HI) in rice is defined as the ratio of grain yield (GY) to biomass (BM). Although it has been demonstrated that HI is significantly related to yield and is considered as one of the most important traits in high-yielding rice breeding, HI-based high-yielding rice breeding is difficult due to its polygenic nature and insufficient knowledge on the genetic basis of HI. Therefore, searching for rice varieties with high HI and mapping genes associated with high HI can facilitate marker-assisted breeding for high HI in rice.

**Results:**

Yuexiangzhan, a popular *indica* cultivar with good reputation of high HI was crossed with Shengbasimiao, an *indica* cultivar with lower HI to develop a recombinant inbred line population, and QTL mapping for HI and its component traits was conducted. In total, five QTLs for HI, three QTLs for GY, and six QTLs for BM were detected in two-year experiments. Among the three GY QTLs, one co-located with the HI QTL on chromosome 8, while the other two co-located with the two tightly-linked BM QTLs on chromosome 3. The co-located QTLs in each of the chromosomal regions produced additive effects in the same direction. Particularly, the HI QTL on chromosome 8, *qHI-8*, could be detected across two years and explained 42.8% and 44.5% of the phenotypic variation, respectively. The existence of *qHI-8* was confirmed by the evaluation of the near isogenic lines derived from a residual heterozygous line, and this QTL was delimitated to a 1070 kb interval by substitution mapping.

**Conclusion:**

In the present study, the detected GY QTLs overlapped with both HI QTL and BM QTL, suggesting a positive relationship between GY and HI or BM, respectively. With an understanding of the genetic basis for grain yield, harvest index and biomass, it is possible to achieve higher yield through enhancing HI and BM by pyramiding the favorable alleles for the two traits via marker-assisted selection (MAS). As *qHI-8* has a large phenotypic effect on HI and expresses stably in different environments, it provides a promising target for further genetic characterization of HI and MAS of high HI in rice breeding.

**Electronic supplementary material:**

The online version of this article (10.1186/s12284-017-0183-0) contains supplementary material, which is available to authorized users.

## Background

Global food demand is increasing with the growth of the world population. Rice is the most important food crop, serving as the staple food for more than half of the world’s population. By 2035, a 26% increase (176 million tons) in rice production will be necessary to feed the growing population (Khush 2013). However, the area available for rice planting in major production countries has been decreasing because of acceleration of urbanization and industrialization. Therefore, increasing the yield per unit area on existing land is the only way to increase rice production.

Harvest index (HI) is an important trait associated with yield. HI is defined as the ratio of grain yield (GY) to biomass (BM), and is taken as a measure of biological success in partitioning photosynthate to the harvestable product (Donald and Hamblin 1976; Sinclair 1998). In the past decades, dramatic increases in crop yield have been largely attributed to improvement in HI (Sinclair 1998). Before the Green Revolution, rice varieties were tall and leafy with weak stems, and had a HI of approximately 0.3. The incorporation of the dwarfing gene *sd1* resulted in increasing HI to 0.5, leading to dramatic increases in rice yield (Khush 2001; Mann 1999). Higher HI is considered to be one of the most important traits in the breeding program for new plant type at the International Rice Research Institute (IRRI) (Khush 1995) and for super hybrid rice in China (Yuan 2001). Although HI is an emphasized trait, rice breeding for high HI is difficult due to the trait’s polygenic nature and insufficient knowledge of its genetic basis. Since the advent of molecular marker technology, some quantitative trait loci (QTLs) for HI have been identified and mapped in rice. Using a double-haploid (DH) population of an *indica*-*japonica* cross, Hittalmani et al. (2003) detected eleven QTLs associated with HI on chromosomes 1, 3, 4, 7 and 8 at nine locations in Asia. Among these QTLs, seven were detected in two to four locations, while the others were only detected in one location. Zhang et al. (2004) identified four QTLs for HI on chromosomes 1, 4, 8 and 11 using a DH population of another *indica*-*japonica* cross. Marri et al. (2005) detected one QTL for HI on chromosome 2 in an interspecific BC_2_ testcross progeny. Sabouri et al. (2009) mapped five QTLs for HI on chromosomes 2, 3, 4 and 5 in the F_2_ population of an *indica-indica* cross. Through association mapping, Li et al. (2012) identified nine markers associated with HI on chromosomes 1, 2, 4, 8 and 9 in Arkansas and Texas, USA, but no common associated marker was found at two locations. Although some QTLs associated with HI have been identified, successful marker-assisted breeding for high HI in rice has not been reported. This issue may be attributed to the rice varieties for studies and the complexity of the trait. In previous studies, the rice varieties used to develop mapping populations have a HI of less than 0.5, and were thus not the ideal sources for genetic studies and high HI breeding. Furthermore, HI is a quantitative trait controlled by multiple genes (QTLs) and the expression of QTL for HI is affected by environments (Hittalmani et al. 2003; Li et al. 2012). Therefore, screening additional rice varieties with higher HI and identifying stably expressed QTLs with large effect on HI will facilitate marker-assisted breeding for high HI in rice.

Yuexiangzhan (YXZ), an *indica* cultivar developed by Rice Research Institute of Guangdong Academy of Agricultural Sciences, China, is an elite variety with high-yield and good quality. Particularly, this variety possesses a HI of 0.616 (Chen et al. 1999). To dissect the genetic basis of HI and its component traits, a recombinant inbred line (RIL) population was developed by crossing YXZ with Shengbasimiao (SBSM), an *indica* cultivar with lower HI, and QTL analysis was conducted to identify and map QTLs for HI, GY and BM in the present study. A novel major QTL for HI, *qHI-8*, could be detected in two-year experiments, and explained 42.8% and 44.5% of the phenotypic variations, respectively. The existence of *qHI-8* and its phenotypic effect on HI was validated and quantified using the near isogenic lines (NILs) derived from a residual heterozygous line (RHL). The results enhance our understanding on the genetic basis of HI in rice. In particular, identification of *qHI-8*, the stably expressed and large effect QTL, provides a promising target for further genetic characterization and marker-assisted breeding for high HI in rice.

## Results

### Harvest indexes of the two parents and their derived RIL population

**Fig. 1:**
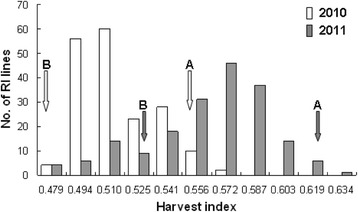
Distribution of harvest index in RIL population derived from YXZ and SBSM in two years (2010 and 2011). A and B indicate the harvest index of YXZ and SBSM, respectively

The two parents, YXZ and SBSM, exhibited significant differences in HI. The HI of YXZ was 0.555 and 0.618 in the years 2010 and 2011, respectively (Table 1). Large HI variations in the RIL population were observed, ranging from 0.486 to 0.568 in 2010, and from 0.471 to 0.642 in 2011 (Table 1). Wider HI variation was observed in 2011 compared to that in 2010 (Fig. 1). Some RI lines (3.2% in 2010 and 1.6% in 2011) had a HI higher than that of YXZ in both years, while some RI lines (17.2%) had a HI lower than that of SBSM in 2011, indicating different transgressive segregation in two-year experiments. The RIL population displayed a continuous and normal distribution for HI (Table 1 and Fig. 1), suggestive of quantitative inheritance and the involvement of multiple genes for the trait. Furthermore, a significantly positive correlation was observed between the HI measured in 2010 and 2011 in the RIL population (*r* = 0.6031, *p* < 0.0001).

**Table 1:** The harvest index of YXZ, SBSM and their derived RIL population in the two-year experiments

Year	Parent		RIL population			
	YXZ (Mean ± SD)	SBSM (Mean ± SD)	Mean ± SD	Range	Skewness	Kurtosis
2010	0.555 ± 0.001****	0.472 ± 0.001	0.515 ± 0.020	0.486–0.568	0.768	−0.437
2011	0.618 ± 0.009***	0.531 ± 0.007	0.563 ± 0.032	0.471–0.642	−0.625	0.089

*** and ****represent the significance of difference between the two parents at *p* < 0.001 and *p* < 0.0001, respectively based on *t*-test

### QTLs for harvest index, grain yield and biomass in RIL population

**Fig. 2:**
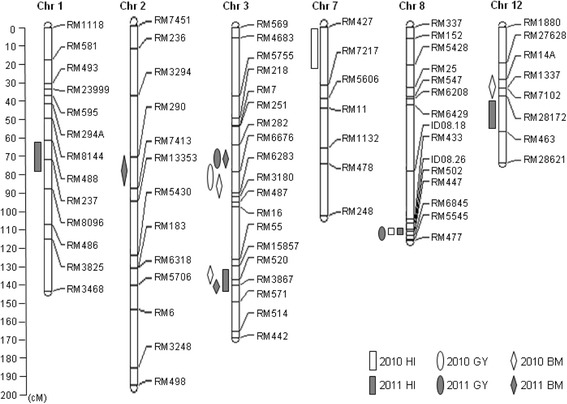
Chromosomal locations of QTLs for harvest index, grain yield and biomass identified in two years (2010 and 2011)

For harvest index, a total of five QTLs were detected and mapped on chromosomes 1, 3, 7, 8 and 12 in two years (Table 2 and Fig. 2). Among the five QTLs, *qHI-7* was detected in 2010 and explained 7.3% of the phenotypic variation, while *qHI-1*, *qHI-3* and *qHI-12* were detected in 2011 and could explain phenotypic variations ranging from 4.6% to 7.4%. It is noteworthy that the QTL on chromosome 8, *qHI-8*, could be detected across both years and could explain 42.8% and 44.5% of the phenotypic variation, with a high LOD of 25.1 and 29.2, in 2010 and 2011, respectively. All positive alleles contributing to HI came from the high HI parent YXZ.

**Table 2:** The QTLs for harvest index, grain yield and biomass identified in RIL population derived from YXZ and SBSM in two years

Trait^a^	QTL	Chr	2010					2011				
			Interval	Interval distance (cM)	LOD	Additive effect^b^	Variation explained (%)	Interval	Interval distance (cM)	LOD	Additive effect	Variation explained (%)
HI	*qHI-1*	1						RM488-RM237	10.4 (6.0)^c^	3.8	0.0069	4.6
	*qHI-3*	3						RM15857-RM520	7.7 (6.0)	5.8	0.0089	7.4
	*qHI-7*	7	RM427-RM7217	31.6 (10.0)	3.2	0.0053	7.3					
	*qHI-8*	8	RM447-RM6845	2.6 (0)	25.1	0.0129	42.8	RM447-RM6845	2.6 (0)	29.2	0.0216	44.5
	*qHI-12*	12						RM28172-RM463	19.4 (10.0)	4.0	0.0082	6.3
GY	*qGY-3a*	3						RM282-RM6676	15.0 (8.0)	11.6	−2.4719	28.0
	*qGY-3b*	3	RM6676-RM6283	11.4 (2.0)	5.8	−1.3457	12.9					
	*qGY-8*	8						RM447-RM6845	2.6 (2.0)	6.5	1.5962	12.1
BM	*qBM-2*	2						RM290-RM7413	16.7 (7.4)	3.5	−2.2656	9.2
	*qBM-3a*	3						RM282-RM6676	15.0 (8.0)	11.0	−4.1157	28.9
	*qBM-3b*	3	RM6676-RM6283	11.4 (8.0)	4.6	−2.4995	10.9					
	*qBM-3c*	3	RM15857-RM520	7.7 (6.0)	3.2	−2.1424	7.2					
	*qBM-3d*	3						RM3867-RM571	9.1 (0)	3.5	−1.9126	6.3
	*qBM-12*	12	RM7102-RM28172	4.4 (2.0)	3.0	−1.8650	6.0					

^a^ *HI* Harvest index, *GY* Grain yield, *BM* Biomass

^b^Positive values indicate effects of allele from YXZ, negative values indicate effects of allele from SBSM

^c^Numbers in parenthesis indicate genetic distance from the LOD peak position to the left marker on the interval

For grain yield and biomass, three and six QTLs were detected, respectively (Table 2 and Fig. 2). Among the three GY QTLs, *qGY-3b* was detected in 2010, while *qGY-3a* and *qGY-8* were detected in 2011. The positive allele at *qGY-8* came from YXZ, while the positive allele at *qGY-3a* and *qGY-3b* came from SBSM. Among the six BM QTLs, *qBM-3b*, *qBM-3c* and *qBM-12* were detected in 2010, while *qBM-2*, *qBM-3a* and *qBM-3d* were detected in 2011. The positive alleles contributing to BM at these QTLs came from SBSM.

Comparing chromosomal locations of the QTLs for GY, HI and BM identified in this study, we found that one GY QTL, *qGY-8*, co-located with the major HI QTL, *qHI-8*, in the interval RM447-RM6845 on chromosome 8; they produced their additive effects in the same direction. The other two tightly linked GY QTLs, *qGY-3a* and *qGY-3b*, co-located with two tightly linked BM QTLs, *qBM-3a* and *qBM-3b*, on chromosome 3. These co-located GY and BM QTLs also produced additive effects in the same direction.

No significant digenic epistatic interaction between the QTLs, nor between the QTLs and other loci, were found (*p* > 0.05) in the present study based on analysis using QTLNetwork-2.0.

### Validation and delimitation of *qHI-8* using NILs derived from a residual heterozygous line

**Fig. 3:**
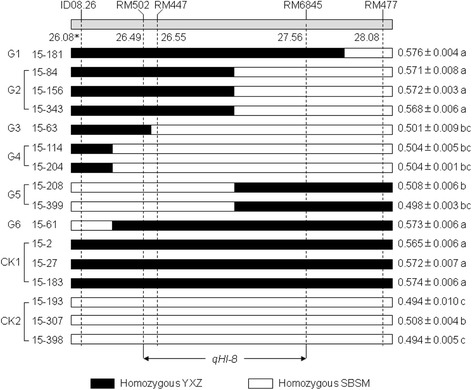
Delimitation of *qHI-8* using the homozygous recombinants. The *qHI-8* was delimited to a 1070 kb interval defined by markers RM502 and RM6845. G1 to G6 indicate the recombinants used in this study, consist of six genotypes according to their positions of recombinant breakpoints and allelic composition. Values in the right side are the HI phenotypes of each recombinant and the controls, CK1 and CK2 (mean ± SD). The values with the same letter indicate no significant difference in HI at *p* = 0.05 based on Duncan’s multiple range test. * The physical position (Mb) of markers on the chromosome

The mapping results reveal that *qHI-8* is a stably expressing and large-effect QTL for harvest index. Additionally, *qHI-8* co-located with *qGY-8,* the QTL for grain yield and they produce their additive effects in the same direction, suggesting its positive effect on grain yield. We believe that *qHI-8* has great potential in high-yielding rice breeding. To validate *qHI-8*, NILs were developed from a residual heterozygous line (RHL). Based on the results of QTL mapping, we searched for RHLs from the 186 RI lines using the markers defining the QTL region of *qHI-8*. One RI line exhibiting heterozygous in the region of *qHI-8* was found. A single plant (RHL15) from this RI line was selected; it is heterozygous in the region harboring *qHI-8* (ID08.26-RM477), but homozygous in other genomic regions based on the genotypes determined by the 142 polymorphic SSR and InDel markers distributed across the 12 rice chromosomes (Additional file 1: Figure S1). RHL15 selfed to produce progenies segregating at the target region in a near isogenic background (equivalent to F_2_, designated as NIL-F_2_). The NIL-F_2_ plants were screened with the polymorphic markers ID08.26, RM502, RM447, RM6845, and RM477 to identify the homozygotes and recombinants within the target region. Based on the genotypes determined by the five markers, three plants (15–2, 15–27 and 15–183) with homozygous YXZ genotypes and three plants (15–193, 15–307 and 15–398) with homozygous SBSM genotypes were selected. They were grouped as two sets of contrasting NILs with and without *qHI-8*, and designated as NIL15-YXZ and NIL15-SBSM, respectively (Table 3 and Fig. 3). Then, NIL15-YXZ, NIL15-SBSM, and the identified recombinant plants further selfed to generate their corresponding F_3_ families. Ten informative homozygous recombinants were identified by genotyping F_3_ individuals with the five markers. These recombinants can be grouped into six genotypes (G1 to G6) according to their positions of recombinant breakpoints and allelic composition (Fig. 3). They have the same genetic background as NIL15-YXZ and NIL15-SBSM except for the target regions, and NIL15-YXZ and NIL15-SBSM were used as controls (CK1 and CK2) to measure HI, respectively. The t-test revealed that no significant differences in HI were observed within lines in NIL15-YXZ or NIL15-SBSM (*p* > 0.05), but highly significant difference in mean HI was observed between NIL15-YXZ and NIL15-SBSM (*p* < 0.0001). The lines carrying *qHI-8* showed consistently higher HI, an average of 0.570, while the lines without *qHI-8* showed consistently lower HI, an average of 0.499 (Table 3). Through HI comparison between NIL15-YXZ and NIL15-SBSM, we could confirm the existence of *qHI-8*. The presence of *qHI-8* resulted in a 0.071 increase in HI.

**Table 3:** Estimation of the relative effect of *qHI-8* on harvest index using two sets of contrasting NILs

NILs	Lines	*qHI-8* ^a^	Harvest index	Average of harvest index	Difference^b^
NIL15-YXZ	15–2	+	0.565 ± 0.006		
	15–27	+	0.572 ± 0.007	0.570	
	15–183	+	0.574 ± 0.006		
					0.071****
NIL15-SBSM	15–193	–	0.494 ± 0.010		
	15–307	–	0.508 ± 0.004	0.499	
	15–398	–	0.494 ± 0.005		

^a^+ and - indicate the presence and absence of *qHI-8* in the lines, respectively

^b^Difference in the average HI between NIL15-YXZ and NIL15-SBSM

****represents the significance of difference in the average HI between NIL15-YXZ and NIL15-SBSM at *p* < 0.0001 based on *t*-test

To further delimitate the *qHI-8* region, differences in HI among recombinant lines and controls (CK1 and CK2) were analyzed using Duncan’s multiple range test. All recombinant lines in G1, G2, and G6 were significantly different from CK2 (*p* < 0.0001), but not significantly different from CK1 (*p* > 0.05), suggesting that *qHI-8* is located within the interval of ID08.26-RM6845. Furthermore, all recombinant lines in G3, G4, and G5 were significantly different from CK1 (*p* < 0.0001), but not significantly different from CK2 (*p* > 0.05), suggesting that *qHI-8* is not located in the intervals of ID08.26-RM502 and RM6845-RM477. Thus, we can delimitate *qHI-8* to a 1070 kb region flanked by RM502 and RM6845 on chromosome 8 (Fig. 3).

## Discussion

### Rice harvest index is an environment-sensitive quantitative trait

Although HI is considered as an important trait closely related to yield in high-yielding rice breeding (Khush, 1995; Yuan, 2001), its genetics is poorly studied compared to other yield-related traits. To better understand the genetic basis of HI in rice, we evaluated the HI of the RIL population and mapped the QTLs for HI in two-year experiments. Our results showed that the HI exhibited continuous and normal distribution in the RIL population, and a total of five QTLs for HI were detected. These results suggest that HI is a quantitative trait controlled by multiple genes (QTLs). Although a good correlation was observed between the HI measured in 2010 and 2011 in the RIL population (*r* = 0.6031, *p* < 0.0001), wider variation of HI among RI lines and more QTLs for HI were detected in 2011compared to that in 2010 (Fig. 1 and Table 2). These differences are mainly attributed to different environments in 2010 and 2011, suggesting that the expression of QTL for HI is affected by the environment. Furthermore, the HI for most of the RI lines fall within the range of their parental values; however, some of RI lines (17.2% in 2011) had lower HI than the low HI parent SBSM, and a few lines (3.2% in 2010 and 1.6% in 2011) had higher HI than the high HI parent YXZ. The recombination of the genetic factors associated with HI in the two parents is one of the main reasons causing transgressive segregation. Even though SBSM has a low HI, there may be undetected alleles with smaller phenotypic effects that are responsible for its HI. Thus, it is possible that some lines carry more favorable combinations of alleles from their parents and have higher HI compared to the high HI parent YXZ, while some lines carry less favorable combinations of alleles and have lower HI compared to the low HI parent SBSM. Transgressive segregation was also observed in the previous studies on yield and its component traits in rice (Hittalmani et al. 2003; Zhang et al. 2004; Liu et al. 2010). Since HI is controlled by multiple genes and the gene expression is affected by the environments, it is necessary to identify the major-effect and stably expressed QTL in order to achieve successful MAS of HI in rice breeding.

### Both harvest index and biomass contribute to grain yield

In the present study, the mapping results showed that the three QTLs for GY, *qGY-8* co-located with the major QTL for HI, *qHI-8* on chromosome 8, the other two tightly linked QTLs, *qGY-3a* and *qGY-3b*, co-located with the two tightly linked QTLs for BM, *qBM-3a* and *qBM-3b* on chromosome 3. The co-located QTLs in each of the chromosomal regions produced their additive effects in the same direction, which was in agreement with significantly positive correlation between GY and HI (*r* = 0.4084, *p* < 0.0001), and between GY and BM (*r* = 0.8859, *p* < 0.0001) in the RIL population (Additional file 2: Figure S2). The results suggest that grain yield increase can be achieved by either harvest index improvement or biomass enhancement. That equal attention should be given to harvest index and biomass in cereal crop breeding was proposed by Donald and Hamblin in 1976. It is common attempt to increase rice productivity by enhancing both harvest index and biomass in super-high yield rice breeding programs (Peng et al. 1999; Ying et al. 1998). However, as both harvest index and biomass are quantitative traits, it is difficult and unreliable to select the two traits based on phenotypes. With knowledge of the genetic basis for grain yield, harvest index and biomass, it is possible or a good strategy to achieve higher yield through enhancing harvest index and biomass by pyramiding the favorable alleles for the two traits via MAS.

### The *qHI-8* is a novel QTL for HI and provides a promising target in molecular rice breeding for high HI

In total, five QTLs for HI from YXZ, *qHI-1, qHI-3, qHI-7, qHI-8* and *qHI-12* were detected in the two-year experiments in the present study. Compared with the chromosomal locations of the QTLs for HI identified in previous studies, *qHI-1* and *qHI-8* are reported for the first time in the present study. Our results also showed that different sets of QTLs for HI were detected in the experiments in 2010 and 2011 (Table 2). Similarly, Hittalmani et al. (2003) detected a total of eleven QTLs for HI in rice at nine locations of Asia representing different environments. Among these QTLs, seven were detected in two to four locations, the others were only detected in one location. Through association mapping, Li et al. (2012) identified seven and two SSR markers significantly associated with HI in Arkansas and Texas, USA, respectively, but no common associated marker was found at two locations. Together with our results, this suggests that most of the QTLs for HI in rice are environment-specific. It is notable that the *qHI-8* identified in the present study could be consistently detected in the two-year experiments representing two different environments, with a high LOD score of 25.1 and 29.2, and explaining 42.8% and 44.5% of the phenotypic variation in 2010 and 2011, respectively. Through the evaluation of HI of the contrasting NILs with and without *qHI-8*, we were able to confirm the existence of *qHI-8* and estimate its effect on HI. Furthermore, through substitution mapping, *qHI-8* was delimited to a 1070 kb region flanked by RM502 and RM6845 on chromosome 8 (Fig. 3). The stable expression and large effect of *qHI-8* on HI makes it to be a promising target in molecular breeding for high HI in rice.

### The function of *qHI-8* on HI may be associated with its promotion in sink formation or transportation of assimilation product

HI in rice reflects the capacity of photosynthate translocation from leaves to panicles during the grain filling period. Therefore, it is related to the allocation of assimilation products and the development of grain yield related traits. To have a higher HI, the rice plant should have a larger sink and higher efficiency in transporting photosynthate to the grains. Therefore, the potential role of the gene underlying the QTLs for HI may be related to one or more of these traits or processes. We recognized that several QTLs related to sink formation and transportation of assimilation product were mapped on the same chromosomal region as the *qHI-8* identified in the present study. These include the QTLs controlling number of primary rachis branches (Nagata et al. 2002; Xu et al. 2004) and number of vascular bundles (Cui et al. 2003; Zhang et al. 2002). Furthermore, *OsSPL16*, a functional gene of the QTL for grain width (Wang et al. 2012) also resides in the region of *qHI-8*. These QTLs/genes located in the same chromosomal region may be the same QTL/gene with pleiotropic effects, crosstalk in the metabolic pathways of sink formation and assimilate transportation or may be the clustering of unrelated genes at this locus. To elucidate if the role of *qHI-8* on increasing HI in rice is due to its positive effect on these traits, we are pursuing fine mapping and cloning of the genes underlying *qHI-8*.

## Conclusion

In the present study, a RIL population derived from a cross betweenYXZ, a popular *indica* cultivar with good reputation of high HI, and SBSM, an *indica* cultivar with lower HI, was used for genetic analysis of HI and its component traits. Our two-year evaluation of HI of the RI lines suggests that the HI in rice is a quantitative trait and affected by the environment. In total, five QTLs for HI, three QTLs for GY, and six QTLs for BM were detected. The detected GY QTLs overlapped with the HI QTL and the BM QTL, suggesting the positive relationships between GY and HI and between GY and BM, respectively. With knowledge of the genetic basis for grain yield, harvest index and biomass, it is possible to achieve higher yield through enhancing HI and BM by pyramiding the favorable alleles for the two traits via MAS. Importantly, the major QTL for HI, *qHI-8*, is reported for the first time in the present study. This QTL explained more than 40% of the phenotypic variation and stably expressed in the two-year experiments. The existence of *qHI-8* was confirmed using NILs, and this QTL was delimitated to a 1070 kb interval flanked by RM502 and RM6845. We believe that the identification of the stably expressed and large-effect *qHI-8* provides a promising target for further genetic characterization of HI and MAS of high HI in rice breeding.

## Methods

### Plant materials

YXZ (high HI) was crossed with SBSM (low HI) and advanced from F_2_ to F_8_ by single seed descent. The resulting RIL population consisting of 186 RI lines was used for QTL analysis in the present study. NILs derived from a residual heterozygous line (RHL15) were used to validate and estimate the relative phenotypic effect of the major QTL for HI, *qHI-8*.

### Trait measurements

The RIL population and their parents were planted in the paddy field equipped with bird-net at the experimental base of Guangdong Academy of Agricultural Sciences in Guangdong, China, in the second cropping season in 2010 and 2011. The experiments were arranged in a randomized complete block design with three replicates. Seeds were sown in the seedling nursery and twelve 20-day-old seedlings of each line were transplanted into one row in the field with a space of 20 cm × 20 cm. The field management, including irrigation, fertilization, and disease and pest control, followed the conventional practice in rice production. At complete maturity, ten plants in the middle of each row were harvested individually at ground level. After drying to constant weight, grain yield (GY; filled grain weight per plant, g) and biomass (BM; filled grains, unfilled grains and straw, g) were measured. HI was calculated as the ratio of GY to BM. Trait measurements averaged over the three replicates in each year were used as the data in QTL analysis.

To validate the existence of the major QTL for HI, *qHI-8*, and estimate its phenotypic effect, HI of the 10 homozygous recombinants and two sets of contrasting NILs, NIL15-YXZ and NIL15-SBSM, were assessed at the same experimental base in the first cropping season in 2014. The experiments were arranged in a randomized complete block design with three replicates. Seeds were sown in the seedling nursery and ten 35-day-old seedlings of each line were transplanted into one row in the field with a space of 20 cm × 20 cm. Eight plants in the middle of each row were harvested individually to evaluate HI as described above.

### DNA extraction and marker analysis

Rice genomic DNA from young seedling was extracted using a modified method described by Dellaporta et al. (1983). For genotyping, simple sequence repeat (SSR) markers evenly distributed over the 12 chromosomes Temnykh et al. 2001; McCouch et al. 2002; International Rice Genome Sequencing Project 2005) and InDel markers designed based on the insertion-deletion polymorphism between YXZ and SBSM were used. PCR reaction was performed in a total volume of 15 ul, containing 15 ng genomic DNA, 0.25 uM each of SSR or InDel primer, 200 uM dNTP mix, 1× PCR buffer, and 0.5 U of Taq polymerase (TaKaRa, Japan) using a Thermal Cycler (DNA Engine, Bio-Rad Laboratories, Inc., USA). The PCR protocol consisted of an initial denaturation at 94 °C for 4 min, followed by 35 cycles of denaturation at 94 °C for 30 s, annealing at 55 °C (50 °C or 61 °C depending on primer sequence) for 30 s, extension at 72 °C for 1 min, and a final extension at 72 °C for 8 min. The PCR products were separated on nondenaturing 8% polyacrylamide gels and stained with Goldview. The gels were documented by a molecular imager (Gel Doc XR, Bio-Rad Laboratories, Inc., USA) for band scoring.

### Construction of linkage map

The linkage map based on the RIL population of 186 lines was constructed using Mapmaker/Exp. 3.0 (Lincoln et al. 1993) with the Kosambi function. A genetic map consisting of 142 markers, covering 12 rice chromosomes and spanning 1427 cM (with the average distance between two markers of 10 cM) was used for QTL analysis.

### Data analysis

The *t*-test, correlation analysis and Duncan’s multiple range test were conducted using SAS program (SAS Institute, 2000).

Composite interval mapping (CIM) was conducted to map QTL by using Windows QTL Cartographer 2.5 (Wang et al. 2007). A LOD threshold of 3.0 was used to declare main-effect QTL. The 1-LOD confidence intervals for the positions of the QTL were also defined based on the CIM results. Digenic epistatic interactions were analyzed using QTLNetwork-2.0 (Yang et al. 2005). The nomenclature of QTL was as described by McCouch et al. (1997).

## Additional files

Additional file 1: Figure S1. Graphical genotype of the RHL15. (DOCX 66 kb)

Additional file 2: Figure S2. Correlation between grain yield and harvest index and between grain yield and biomass. (DOCX 65 kb)

## Abbreviations

BM: Biomass
cM: centi-Morgan
GY: Grain yield
HI: Harvest index
InDel: Insertion-deletion
MAS: Marker-assisted selection
NIL: Near isogenic line
QTLs: Quantitative trait loci
RHL: Residual heterozygous line
RIL: Recombinant inbred line
SBSM: Shengbasimiao
SSR: Simple sequence repeat
YXZ: Yuexiangzhan
