# Supplementary material for: Identification and validation of a novel major QTL for harvest index in rice (Oryza sativa L.)
Source: Rice (N Y). 2017 Sep 26;10:44. doi: 10.1186/s12284-017-0183-0 (PMC5615080; doi:10.1186/s12284-017-0183-0)
Supplement: Supplementary file 1 — Graphical genotype of the RHL15. (DOCX 66 kb) [file 12284_2017_183_MOESM1_ESM.docx]

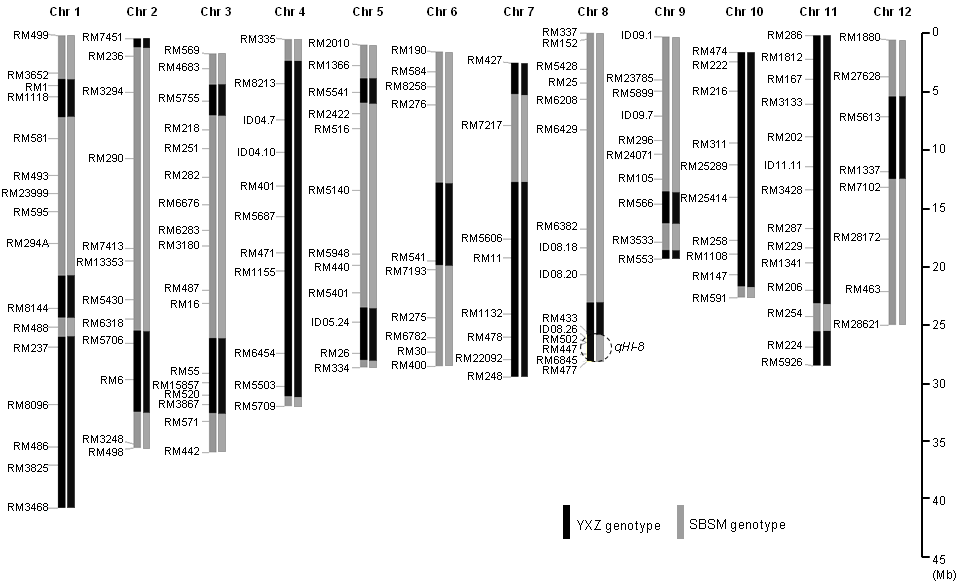


Figure S1 Graphical genotype of the RHL15

RHL15 is heterozygous in the region harboring *qHI-8* (ID08.26-RM477), but homozygous in other genomic regions based on the genotypes determined by the 142 polymorphic SSR and InDel markers distributed across 12 rice chromosomes. The circle indicates the region harboring *qHI-8*.
