# Supplementary material for: Identification and validation of a novel major QTL for harvest index in rice (Oryza sativa L.)
Source: Rice (N Y). 2017 Sep 26;10:44. doi: 10.1186/s12284-017-0183-0 (PMC5615080; doi:10.1186/s12284-017-0183-0)
Supplement: Supplementary file 2 — Correlation between grain yield and harvest index and between grain yield and biomass. (DOCX 65 kb) [file 12284_2017_183_MOESM2_ESM.docx]

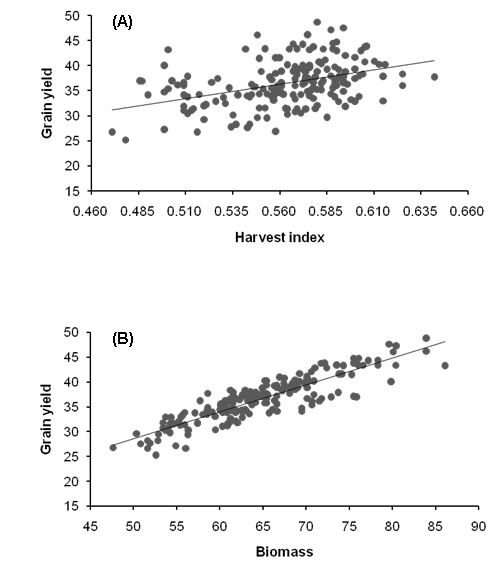


Figure S2 Correlation between grain yield and harvest index (A) , grain yield and biomass (B)

in RIL population
